# Macrophages Facilitate Coal Tar Pitch Extract-Induced Tumorigenic Transformation of Human Bronchial Epithelial Cells Mediated by NF-κB

**DOI:** 10.1371/journal.pone.0051690

**Published:** 2012-12-05

**Authors:** Feifei Feng, Yiming Wu, Shaofeng Zhang, Yu Liu, Lijuan Qin, Yongjun Wu, Zhen Yan, Weidong Wu

**Affiliations:** 1 Department of Occupational and Environmental Health, College of Public Health, Zhengzhou University, Zhengzhou, Henan, China; 2 Department of Sanitary Chemistry, College of Public Health, Zhengzhou University, Zhengzhou, Henan, China; 3 Center for Environmental Medicine, Asthma, and Lung Biology, University of North Carolina at Chapel Hill, Chapel Hill, North Carolina, United States of America; Dartmouth, United States of America

## Abstract

**Objective:**

Chronic respiratory inflammation has been associated with lung cancer. Tumor-associated macrophages (TAMs) play a critical role in the formation of inflammation microenvironment. We sought to characterize the role of TAMs in coal tar pitch extract (CTPE)-induced tumorigenic transformation of human bronchial epithelial cells and the underlying mechanisms.

**Methods:**

The expression of TAMs-specific CD68 in lung cancer tissues and paired adjacent tissues from cancer patients was determined using immunostaining. Co-culture of human bronchial epithelial cells (BEAS-2B) and macrophage-like THP-1 cells were conducted to evaluate the promotive effect of macrophages on CTPE-induced tumorigenic transformation of BEAS-2B cells. BEAS-2B cells were first treated with 2.4 µg/mL CTPE for 72 hours. After removal of CTPE, the cells were continuously cultured either with or without THP-1 cells and passaged using trypsin-EDTA. Alterations of cell cycle, karyotype, colony formation in soft agar and tumor xenograft growth in nude mice of BEAS-2B cells at passages 10, 20 and 30, indicative of tumorigenecity, were determined, respectively. In addition, mRNA and protein levels of NF-κB in BEAS-2B cells were measured with RT-PCR and western blot, respectively. B(a)P was used as the positive control.

**Results:**

The over-expression of TAMs-specific CD68 around lung tumor tissues was detected and associated with lung cancer progression. The tumorigenic alterations of BEAS-2B cells including increase in cell growth rate, number of cells with aneuploidy, clonogenicity in soft agar, and tumor size in nude mice *in vivo* occurred at passage 10, becoming significant at passages 20 and 30 of the co-culture following CTPE removal in compared to BEAS-2B cells alone. In addition, the expression levels of NF-κB in BEAS-2B cells were positively correlated to the malignancy of BEAS-2B cells under different conditions of treatment.

**Conclusion:**

The presence of macrophages facilitated CTPE-induced tumorigenic transformation of BEAS-2B cells, which may be mediated by NF-κB.

## Introduction

Lung cancer is the leading cause of cancer mortality worldwide [1], with 1.2 million deaths each year. And there are 1.3 million new cases being diagnosed every year in the world. In China, it is predicted from current epidemiological data that 10 million of people may be diagnosed of lung cancer in 2025. However, the overall 5-year survival rate for lung cancer patients is still less than 15%, which has remained largely stable for the last three decades. Lung carcinogenesis is a complex process, and elucidation of the molecular mechanisms involved in the pathogenesis of lung cancer is expected to help develop novel diagnostic and therapeutic strategies against lung cancer.

In 1863 Rudolf Virchow first reported the association of inflammation with cancer [2]. Since then, cancer-related inflammation has been included as a hallmark of carcinogenesis [3]. It has been proposed that tumors were considered as wounds that do not heal because of permanent inflammatory infiltration [4]. Increasing epidemiological evidence has demonstrated that chronic inflammation may play a critical role in lung carcinogenesis [5], [6], [7]. Individuals with chronic inflammatory respiratory diseases, such as chronic obstructive pulmonary disease resulted from smoking exposure [8], and chronic hypersensitivity pneumonitis [9] were at higher risk for subsequent development of lung cancer. Furthermore, the regular use of aspirin and other non-steroidal anti-inflammatory drugs can reduce the risk of lung cancer, not only in animal, but also in human [10], [11], [12], [13]. However, the mechanisms of inflammation-promoted initiation of lung cancer have not been fully understood.

Emerging evidence showed that tumor-associated macrophages (TAMs), derived from circulating monocytic precursors, form a major components in tumor microenvironment. TAMs infiltration has been found in many malignant cancers, such as cervical cancer, colorectal cancer, anaplastic thyroid carcinoma, breast cancer [14], [15], [16], [17], and lung cancer [18], which contributes to angiogenesis, lymphargiogenesis, invasion and metastasis. TAMs represent a first line of cells in promoting tumor development because TAMs can release pro-inflammatory cytokines and form tumor microenvironment, which may support tumor growth and help tumor evade immunosurveillance [19], [20]. So far, no evidence has been reported on the role of TAMs in initiation of lung tumor.

**Figure 1:**
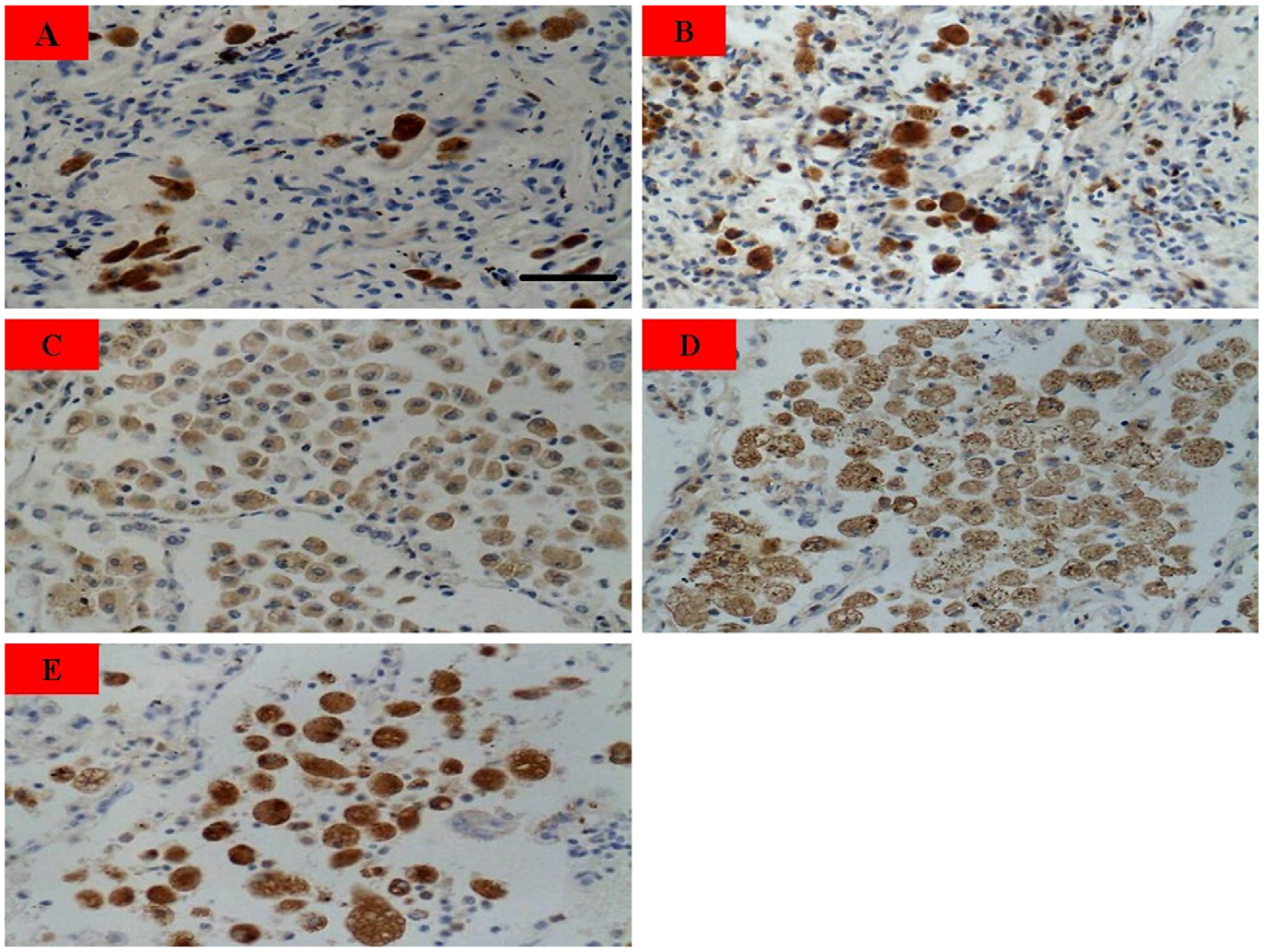
CD68 protein expression levels in lung tumor tissue, adjacent tumor tissue and surrounding non-tumorous lung tissue from lung cancer patients. Scale bar = 20 µm. (**A**) Adenocarcinoma; (**B**) Squamous cell carcinoma; (**C**) adjacent adenocarcinoma tissue; (**D**) adjacent squamous cell carcinoma tissue; (**E**) non-tumor. CD68 were mainly detected in cytoplasm of macrophages with brown staining. Positive cell number of CD68 in adjacent lung cancer tissue (**C–D**) was more than that in lung cancer tissues (**A–B**) and non-tumorous lung tissues (**E**) (n = 67, *P*<0.05).

Coal tar pitch (CTP), the by-product of coal tar incomplete burning and distillation, is used broadly for producing carbon electrode adhesive, waterproof, anti-corrosion coatings and road-construction materials. On daily bases, people are exposed to CTP fume. At present, many studies have evaluated the carcinogenic potential of CTP and proved it as a selective inducer of lung cancer. Weyand and coworkers [21] have reported high lung cancer incidence of female A/J mice following 260 days of CTP administration in the diet. Koganti et al. [22] found that an adduct detected in rat lungs 24 hours after administration of dimethyl-sulfoxide (DMSO) extracted coal tar pitch by i.p. injection for 3 days. In the previous study we have showed that tracheal instillation of 200 µL CTP into rat lungs for 8 times at the concentration of 160 mg/mL, induced lung cancer [23]. CTP is a recognized carcinogen, and CTP-induced lung cancer is one of the serious occupational disorders. Given the long latent period, CTP-induced lung cancer may serve as an excellent model for studying the mechanisms of carcinogenesis.

In this study, human bronchial epithelial cells (BEAS-2B) were utilized as the in *vitro* model to explore the relationship between inflammation and the tumorigenicity induced by coal tar pitch extract. The reason for using bronchial epithelial cells was mainly based on the fact that most lung cancer originates histologically from bronchial epithelial cell. Coal tar pitch extract (CTPE)-treated BEAS-2B cells were cultured in the presence or absence of macrophage-like THP-1 cells to explore the role of macrophages in CTPE-induced tumorigenic transformation of BEAS-2B cells and the underlying mechanisms.

**Table 1:** Association between CD68 expression in adjacent tumor tissue and clinical characteristics of lung cancer (n = 67).

Clinicopathologic characteristics	n	CD68 + cell number	*P*
**Age(y)**			
≥60	30	97.13±2.52	0.299
<60	37	97.78±2.55	
**Sex**			
Male	35	97.13±2.36	0.234
Female	32	97.87±2.71	
**Phatological type**			
Squamous cell carcinoma	38	97.61±2.50	0.642
Adenocarcinoma	29	97.32±2.62	
**Tumor cell differentiation**			
Well & Moderate	50	97.25±2.45	0.200
Poor	17	98.17±2.75	
**TNM stage**			
Ι+Π	42	95.85±1.14	0.001
ΙΙΙ + ΙV	25	100.24±1.72	
**Metastasis to lymph node**			
N0	26	99.87±1.95	0.001
N1	41	95.98±1.50	
**Tumor invasion to Pleura**			
Yes	31	98.71±2.66	0.001
No	36	96.44±1.90	

## Materials and Methods

### Preparation of Coal tar pitch extract (CTPE)

CTPE was collected as described previously [24]. Briefly, moderate-temperature CTP was grinded into powder in an agate bowl, sieved by 200 mesh sieves and heated at 400°C on the electric hot plate in a flow hood. CTP fume was collected on cellulose ester membrane with 0.8 µm pore size by regular dust sampler and respiratory dust sampler, the particles were collected from diameter 0.8 µm to 2.5 µm. Sampling duration was 100 min with a flow of 10 L/min. After sampling, the filter membranes were cut into pieces, put into flask with stopper and dissolved into 50 mL acetidin (spectroscopically pure), then the solution was vibrated supersonically for 40 min and filtered by sand core funnel, the supernatant was got. Finally a beaker with the supernatant was put in 45°C drying baker, when the liquid was volatilized completely, DMSO (spectroscopically pure) was added to get the extract solution. The stock concentration of CTP extract was 2 mg/mL.

**Figure 2:**
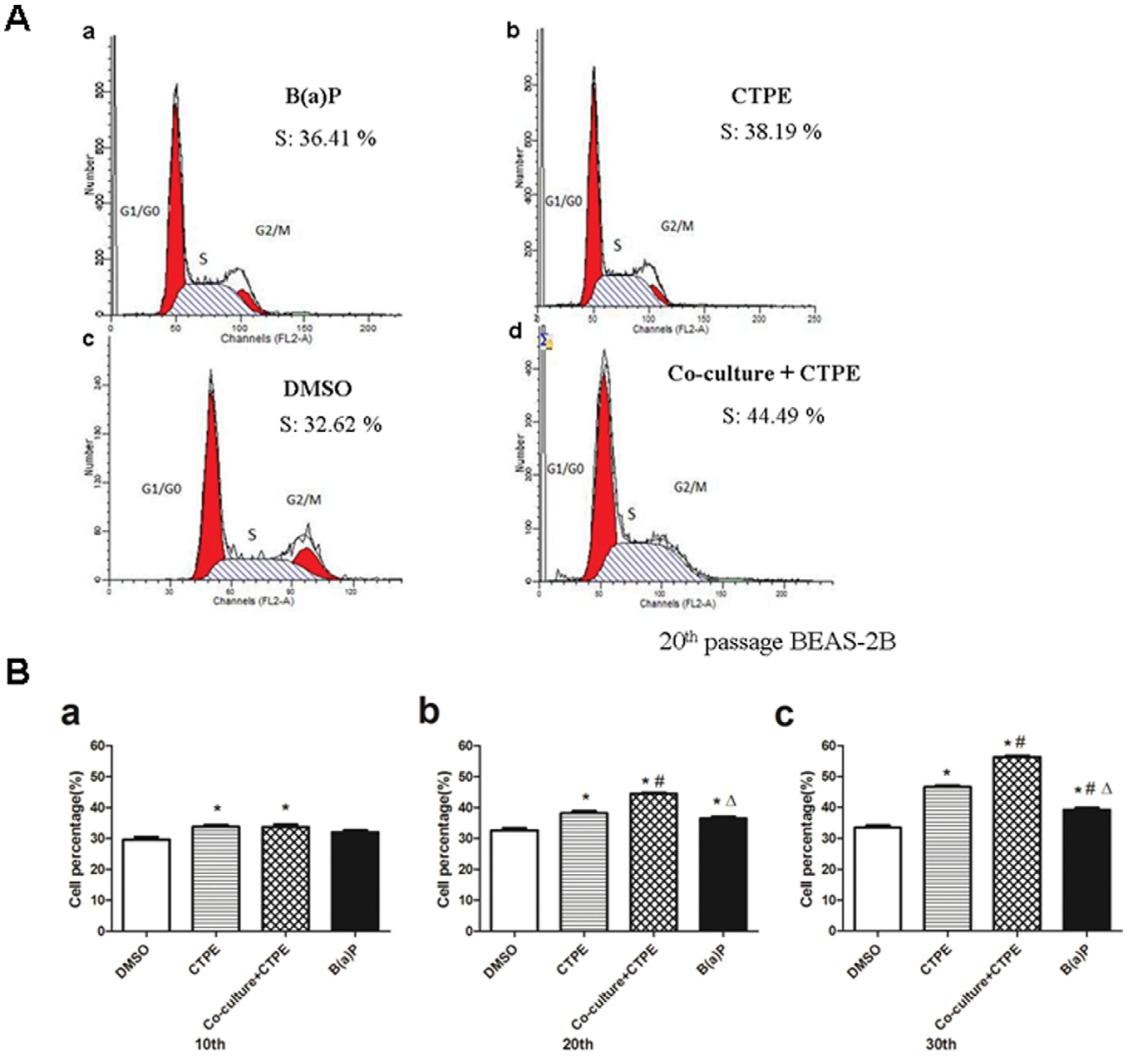
Cell growth rate assays of BEAS-2B cells at passage 10, 20 and 30 in four groups. BEAS-2B cells were treated with 2.4 µg/mL CTPE for 72 hours. After removal of CTPE, the BEAS-2B cells were cultured in the presence or absence of THP-1 cells and passaged. The percentage of S-phase BEAS-2B cells at passage 10, 20 and 30 was determined using flow cytometry. (**A**) Representatives of DNA content of BEAS-2B cells at passage 20 in four groups. (**B**) The percentage of BEAS-2B cell in S phase among different passages and groups. The percentage of the S phase cells in Co-culture/CTPE group started to increase at passage 10; was higher than that of CTPE or B(a)P at passage 20, and kept the highest level at passage 30.(n = 6, *: vs DMSO, *P*<0.05; #: vs CTPE, *P*<0.05; Δ:vs Co-culture+CTPE, *P*<0.05). Phenotypes were triplicated.

**Figure 3:**
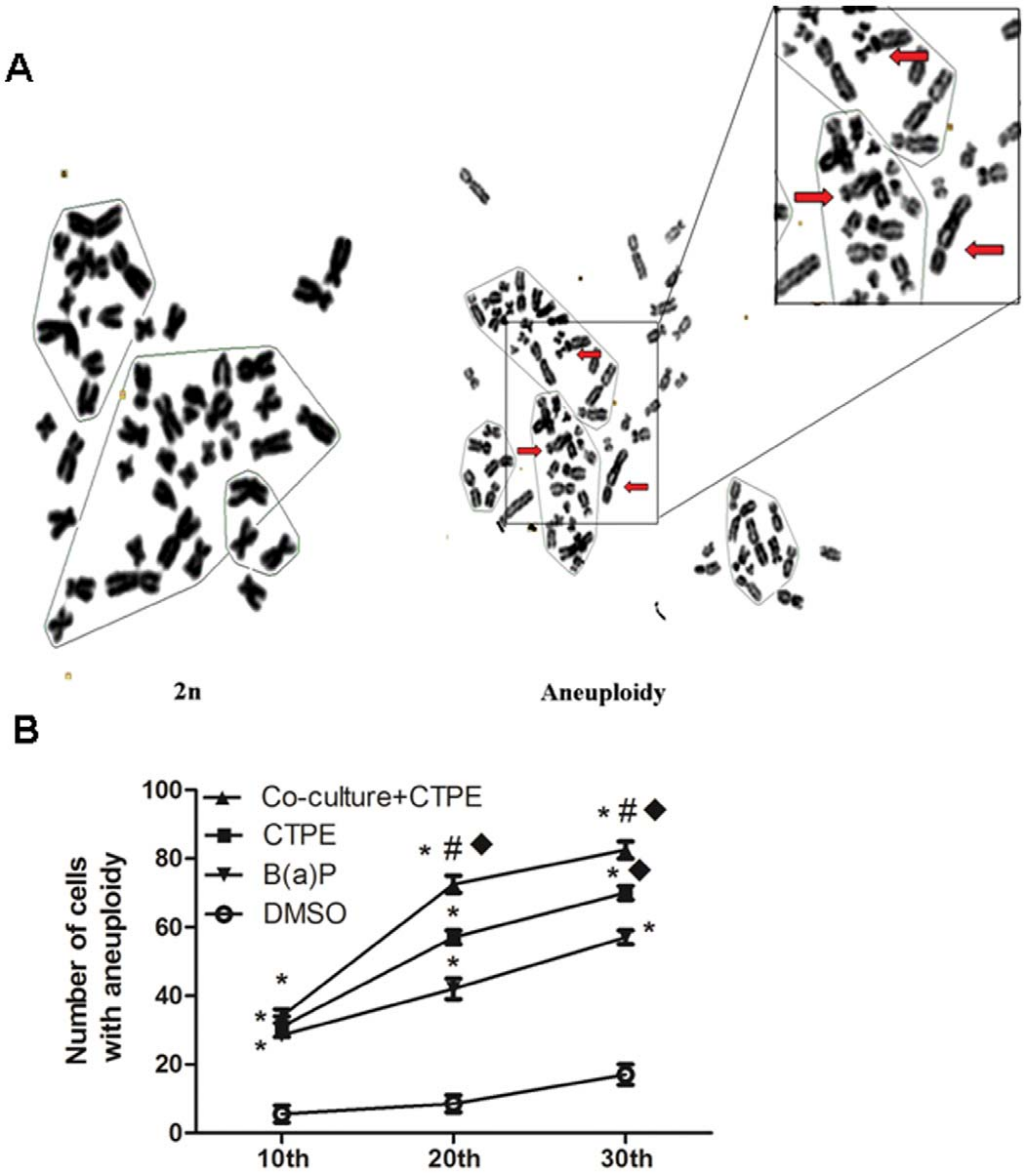
The number of BEAS-2B cells with aneuploidy in Co-culture/CTPE group increased at passage 10, 20 and 30. Aneulpoidy was observed based on abnormal hromosome numbers, including hypodiploid (<2n) and hyperdiploid (>2n∼<4n). (**A**) Representative of normal chromosome (2n) and aneuploidy (>2n). Red arrow showed double centromere. (**B**) The number of cells with aneuploidy in total 100 BEAS-2B cells in Co-culture/CTPE group at passage 10 was more than that of DMSO group, but was not increased compared with CTPE group. At passage 20 and 30, the rates of cells with aneuploidy in 100 BEAS-2B cells in Co-culture/CTPE group were peaked among these four groups. (n = 6, *: vs DMSO, *P*<0.05; #: vs CTPE, *P*<0.05; ♦:vs B(a)P, *P*<0.05.). Phenotypes were triplicated.

### Cell Lines, Cell Culture and Tissue Specimens

Human bronchial epithelial cell line (BEAS-2B): The BEAS-2B cell line (subclone S6) was obtained from Drs, Curtis Harris and John Lechner (USA National Institutes of Health). It was derived by transforming human bronchial epithelial cells with an adenovirus 12-simian virus 40 construct [25]. Human macrophage-like cell line (THP-1) was purchased from ATCC (Rockville, USA). The two cell lines were cultured in RPMI 1640 medium with 10% (v:v) of fetal bovine serum (FBS), 100 IU/mL of penicillin and 100 mg/mL of streptomycin. All the cells were cultured at 37°C in a 5% CO_2_ incubator.

**Figure 4:**
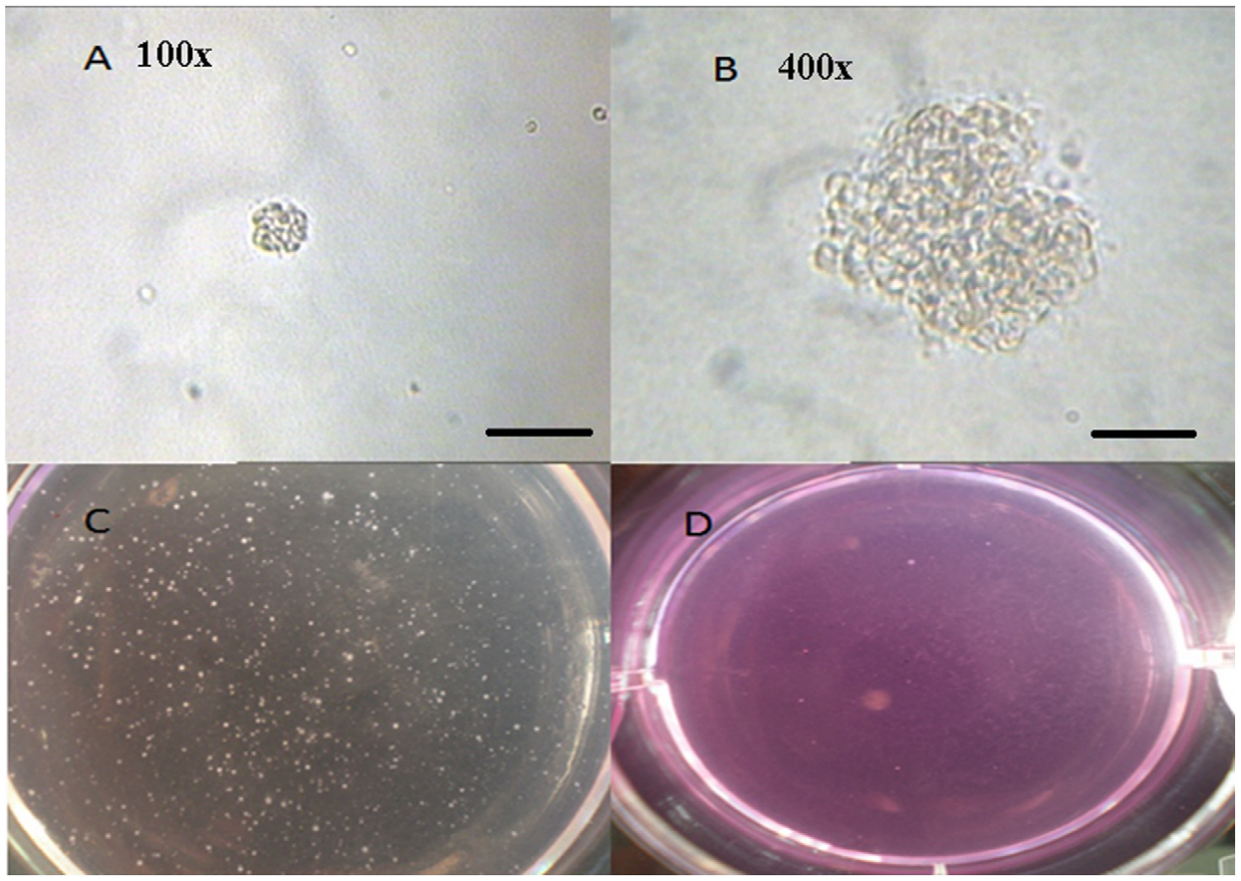
Representatives of colony in soft agar. (**A**) A colony in soft agar, scale bar = 10 µm; (**B**) A colony in soft agar, scale bar = 50 µm; (**C**) The representative of colonies of BEAS-2B cells in Co-culture/CTPE group at passage 20 in dish; (**D**) The representative of colonies of BEAS-2B cells treated with DMSO at passage 20 in dish.

**Figure 5:**
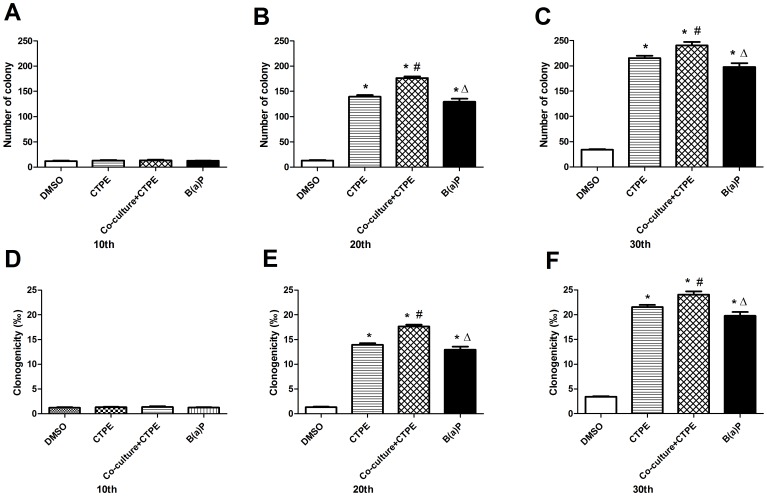
The number of colony and percentage of colonies formation of BEAS-2B cells in soft agar. The number of colony (**A–C**) and clongenicity percentage (**D–F**) were significantly increased in Co-culture/CTPE group compared to those of other three groups at passage 20, or passage 30. (n = 6, *: vs DMSO, *P*<0.05; #: vs CTPE, *P*<0.05; Δ:vs Co-culture+CTPE, *P*<0.05).Values were the mean±SD of three independent experiments.

67 pairs of paraffin-embedded primary lung cancer, their adjacent tumor tissue and surrounding non-tumor lung tissues were collected from patients with lung cancer at the First Affiliated Hospital of Zhengzhou University (Henan, China). Written informed consent was obtained from all 67 subjects. The Life Sciences Institutional Review Board of Zhengzhou University approved the consent procedure.

### Immunohistochemistry (IHC)

Briefly, specimens were deparaffinized, blocked with goat serum for 30 min, and incubated with mouse anti-human CD68 monoclonal antibody (Santa Cruz, 1∶10 dilution) overnight at 4°C, then incubated with biotinylated rabbit anti-mouse immnunoglobulin at a concentration of 1∶100 at 37°C for 30 min. Positive expression of CD68 IHC was reflected as the brown staining in the cytoplasm and estimated by averaging positively-staining cell numbers under 10 high power vision fields.

**Figure 6:**
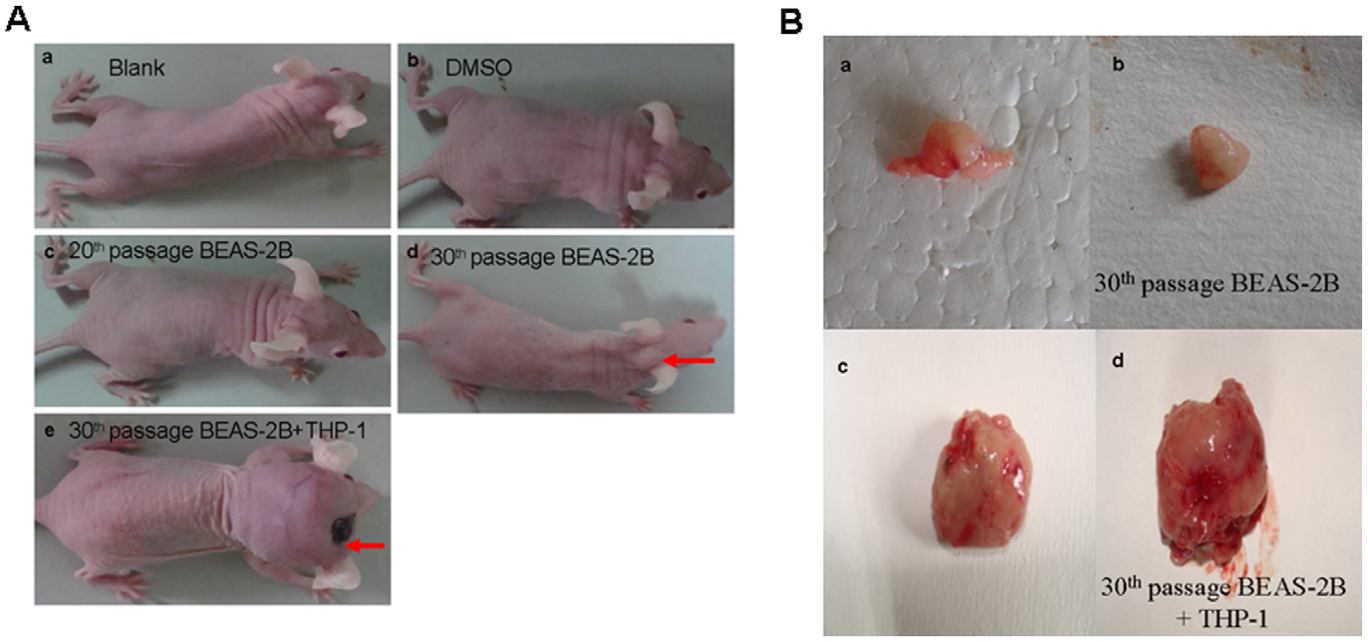
Larger tumor was formed in nude mice transplanted with mixed CTPE-induced BEAS-2B cells at passage 30 and THP-1 cells. (**A**) Representative examples of tumor formation in nude mice on the 30^th^ day after injection (n = 6/group). (**a**) untrested BEAS-2B cells, (**b**) BEAS-2B cells induced with DMSO, (**c, d**) CTPE-induced BEAS-2B cells at passage 20 and 30, (**e**) CTPE-induced BEAS-2B cells at passage 30 mixed with THP-1 cells. Red arrow showed tumors. (**B**) Representatives of tumor removed from nude mice after 30 days transplantion. (**a–b**) CTPE-induced BEAS-2B cells at passage 30, (**c-d**) CTPE-induced BEAS-2B cells at passage 30 mixed with THP-1 cells.

**Figure 7:**
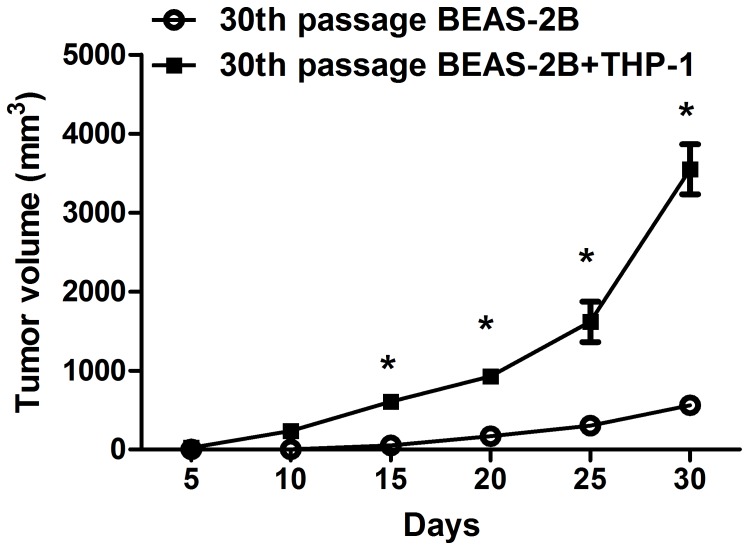
Growth curves of tumor in nude mice. The average volume of tumors derived from CTPE-induced BEAS-2B cells at passage 30 and THP-1 cells increased compared to the CTPE-induced BEAS-2B cells at passage 30 alone at different observation time points (n = 6, *P<0.05).

### Determination of CTPE Half Maximal Inhibitory Concentration (IC_50_)

BEAS-2B cells were placed into 24-well plates at a density of 1×10^4^ per well and treated vehicle control (DMSO), 1, 2.5, 5, 10, 20, 40 and 80 µg/mL of CTPE for 24 h. Then cell viability determined using trypan blue dye exclusion assay. The experiment was repeated three times. And the half maximal inhibitory concentration (IC_50_) was calculated using Probit regression, which was 8.11 µg/mL.

**Figure 8:**
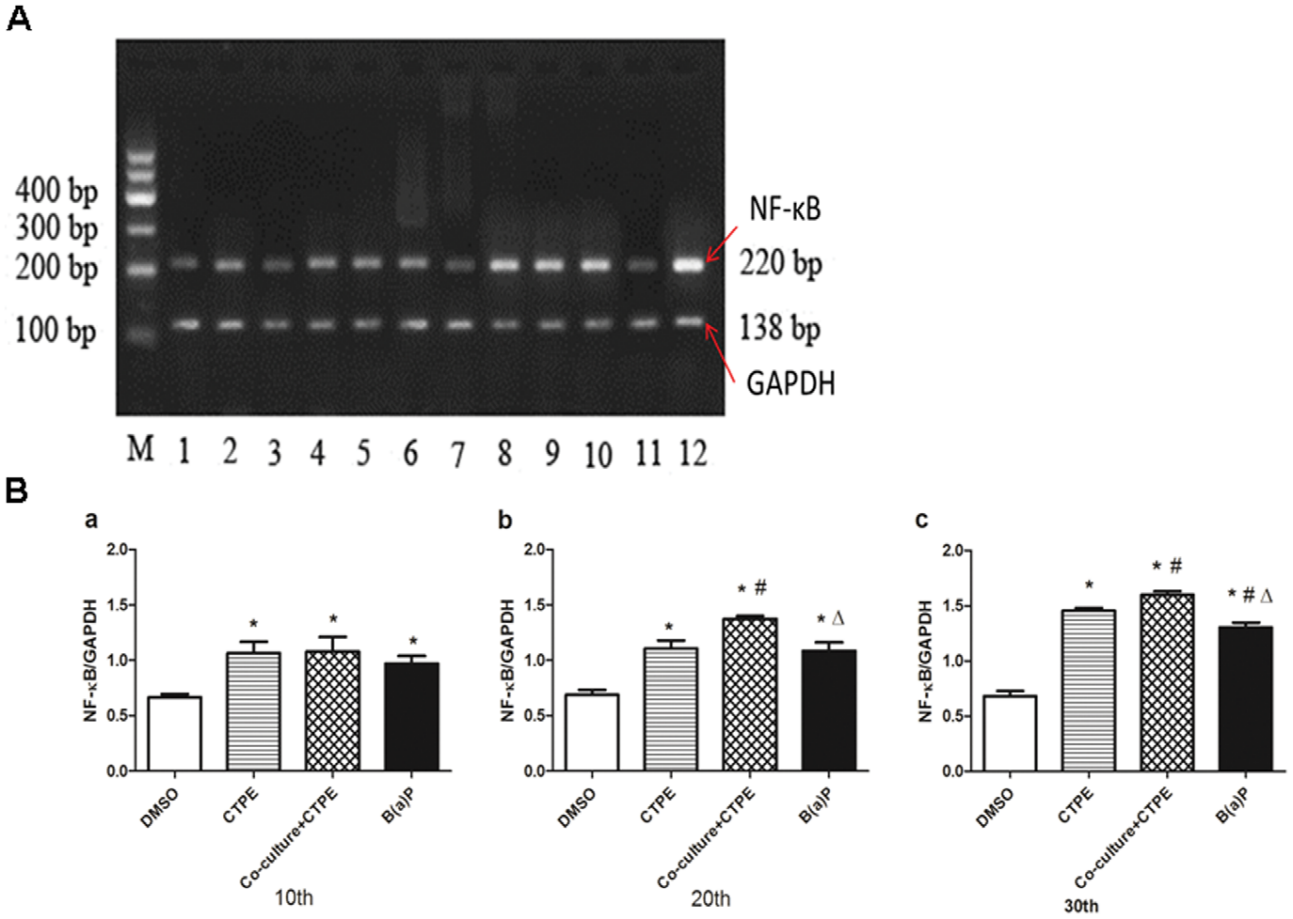
The gene levels of NF-κB in the BEAS-2B cells at passages 10, 20 and 30. (**A**) NF-κB mRNA of BEAS-2B cells in four groups on agarose gel. M:marker;1–4 lanes: 10^th^ B(a)P, 10^th^ CTPE, 10^th^ DMSO, 10^th^ Co-culture+CTPE; 5–8 lanes: 20^th^ B(a)P, 20^th^ CTPE, 20^th^ DMSO, 20^th^ Co-culture+CTPE; 9–12 lanes: 30^th^ B(a)P, 30^th^ CTPE, 30^th^ DMSO, 30^th^ Co-culture+CTPE. (**B**) Quantitative comparison of NF-κB mRNA. The level of NF-κB mRNA in Co-culture/CTPE group started to increase at passage 10, which was higher than that of CTPE or B(a)P at passage 20, and kept the highest level at passage 30.(n = 6, *: vs DMSO, *P*<0.05; #: vs CTPE, *P*<0.05; Δ:vs Co-culture+CTPE, *P*<0.05). The data was from two independent experiments.

**Figure 9:**
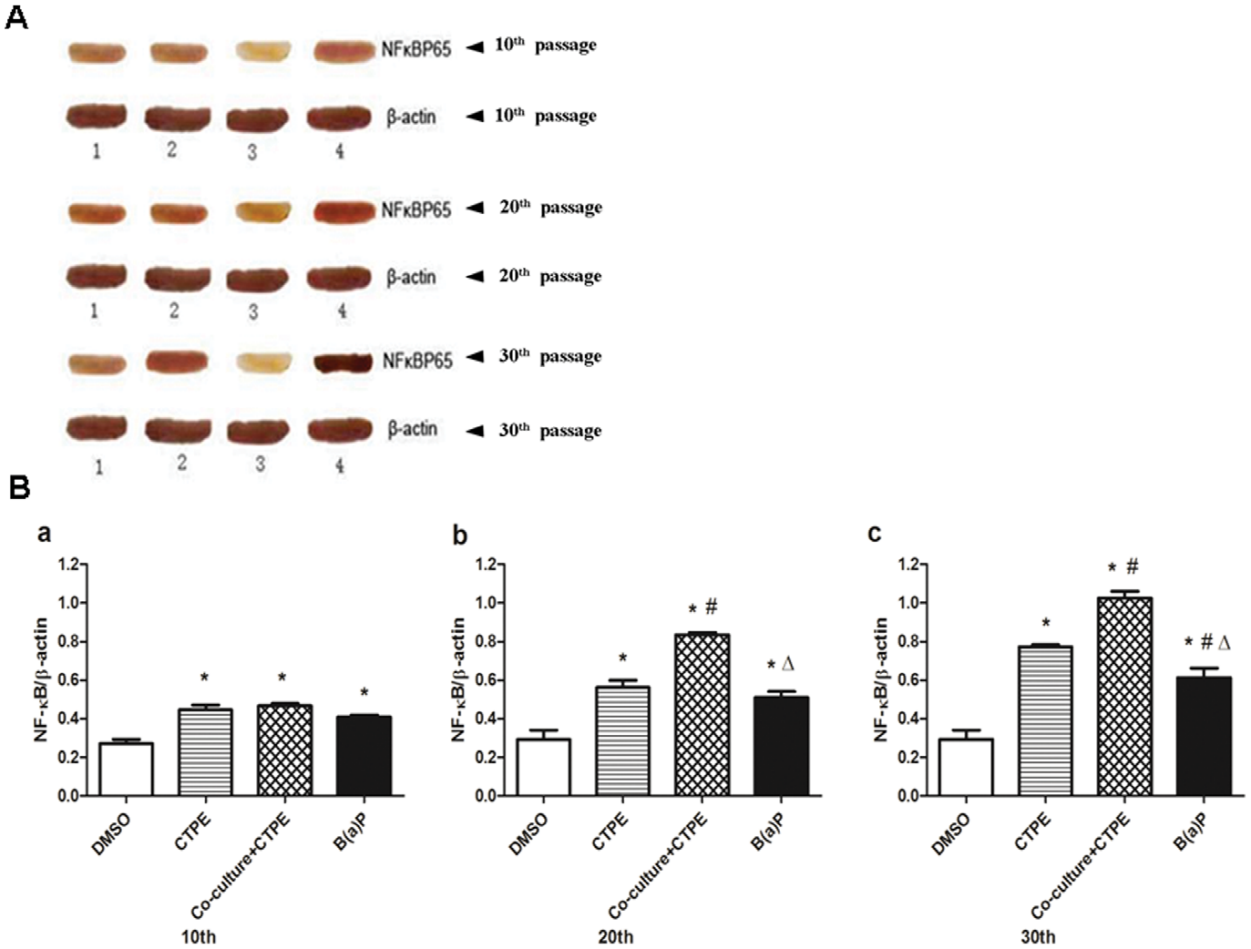
The protein levels of NF-κB in the BEAS-2B cells at passages 10, 20 and 30. (**A**) The protein bands of NF-κB in the BEAS-2B cells at passage 10, 20 and 30 in four groups were shown by Western blot. 1–4 bands: B(a)P, CTPE, DMSO, Co-culture+CTPE. (**B**) Quantitative comparison of the densitometry of NF-κB protein bands intensities normalized to the loading control β-actin. The level of the relative densitometry of NF-κB protein in Co-culture/CTPE group started to increase at passage 10, which was higher than that of CTPE or B(a)P at passage 20, and kept the highest level at passage 30.(n = 6, *: vs DMSO, *P*<0.05; #: vs CTPE, *P*<0.05; Δ:vs Co-culture+CTPE, *P*<0.05). There are representatives from two similar experiments.

### CTP Treatment of Co-culture of BEAS-2B and THP-1 Cells

BEAS-2B cells were treated with 2.4 µg/mL CTPE (30% of IC_50_) for 72 h as follows: BEAS-2B cells grown to 70%–80% confluence were treated with CTPE solution for 24 h. After removal of CTPE, the cells were washed with cold PBS and passaged using trypsin-EDTA; The BEAS-2B cells underwent the same procedure for another two times, total of 72 h. Then, CTPE-treated BEAS-2B cells were co-cultured with THP-1 cells in a ratio of 10∶1(BEAS-2B cells/THP-1 cells).

B(a)P (5 µg/mL) was used as the positive control and 0.1% DMSO as vehicle control. For simplification, the passage of BEAS-2B cell was numbered as passage 0 following CTPE treatment.

### Examination of Cell Cycle with Flow Cytometry

BEAS-2B cells at passages 10, 20 and 30 were fixed in 70% ethanol prior to propidium iodide staining. DNA content was evaluated by FACS flow-cytometry (Shimadzu, Japan).

### Karyotyping

BEAS-2B cells at passages 10, 20, and 30 in logarithmic phase were treated with 0.04 µg/mL colcemid for 3 h at 37°C to arrest cells in metaphase. Then the cells were trypsinized, centrifuged for 10 min at 1000 rpm, and the cell pellets resuspended in warmed (37°C) KCl hypotonic solution and incubated for 40 min. The swollen cells were pelleted and resuspended in 8 mL of Carnoy’s fixation solution (3∶1 = methanol: glacial acetic acid) at room temperature for 2 h. The cell suspension was centrifuged and washed twice in fixation solution. After the last centrifugation, the cell was resuspended in 2 mL freshly prepared fixation solution. Three drops of the final cell suspension were placed on clean slides and air-dried. Slides were stained with Giemsa solution (pH 6.8) for 30 min, washed with tap water for 5 seconds, and air-dried. One hundred cells in metaphase were examined for karyotyping (chromosomal abnormality).

### Colony Formation in Soft Agar

Colony formation in soft agar was performed by growing 1×10^4^ of BEAS-2B cells from passages 10, 20 and 30 in the upper layer (0.7% seaplague) of the two layer agar (0.7% and 1.2%) in a 6-cm dish. After 3 weeks, the number of colony (a colony consisted of more than 50 cells) was counted, and clonogenicity (‰) was calculated as number of colony/total growing cell number×1000‰.

### Tumor Formation in Nude Mice

6 weeks old male nude mice were purchased from Hunan Slack King Laboratory Animal Co., Ltd. (Changsha, China). The experiment protocol of tumor formation in nude mice was approved by the Life Sciences Institutional Review Board of Zhengzhou University. Mice were treated humanely and with regard for alleviation of suffering.

BEAS-2B cells under different conditions (blank control, DMSO treatment, different passages of BEAS-2B cells after CTPE treatment, and the mixture of the CTPE-induced BEAS-2B cells at passage 30 and THP-1 cells (2∶1)) in 200 µL PBS were injected into the back neck of 4 weeks old nude mice (n = 6 for each group). The total cell number inoculated into each mouse was 2×10^7^. The size of the tumor was estimated according to the formula V = L×W^2^/2 every 5 days after inoculation. The tumor was removed and weighted on the 30^th^ day.

### RT-PCR

Total RNA was isolated from BEAS-2B cells using RNAeasy kit (Invitrogen, Carlsbad, CA, USA) following the manufacturer’s protocol. Samples were treated with DNase to remove DNA contamination (Ambion Inc., Austin, TX, USA). RNA samples were then reverse-transcribed into cDNA using SuperScript II RT (Invitrogen Corp., Carlsbad, CA, USA) following the manufacturer’s instruction. Primers were designed using Primer 5.0 software and were produced by Shanghai Biological Engineering Company. The sequences are as follows: NF-κB forward: 5′-TGCCGAGTGAACCGAAAC-3, reverse 5′-TGGAGACACGCACAGGAGC-3′. Gene expression values were normalized to the housekeeping gene GAPDH. PCR reaction contained a total volume of 100 µL containing 1.5 mM magnesium chloride (MgCl_2_), 200 µM deoxynucleoside triphosphate, 2.5 units Taq DNA polymerase, 20 picomoles of primer, and 100 ng cellular DNA templats. Taq PCR master mix kit was used for PCR reactions (Shanghai Xinbainuo Bio. Co., Shanghai, China). The PCR reaction for NF-κB was performed under the following conditions: 95°C for 2 min, 56°C for 30 s, 72°C for 45 s, for 29 cycles. For GAPDH, PCR condition was 95°C for 2 min, 59°C for 1 min, 72°C for 1 min, for 28 cycles. The two PCR products mixed together and were analyzed by 1.5% agarose gels in TBE buffer. The gels were observed and taken pictures using UV imaging equipment. The gray values were analyzed using Gelpro3.5 software.

### Western Blotting

BEAS-2B cells were lysed with lysis buffer (RIPA lysis buffer with proteinase inhibitors). Protein concentration of cell lysate supernatants was determined with Bradford assay. 20 µg protein was mixed with sample loading buffer and boiled for 5 min. Samples were separated by 10% SDS-PAGE and transferred onto PVDF membranes. Immunoblots were blocked with 5% milk in TBS/0.1% Tween 20 for 1 h at room temperature, then incubated overnight at 4°C with 1∶500 rabbit polyclonal antibody against human NF-κB p65 (Santa Cruz) and 1∶1000 rabbit polyclonal antibody to human β-actin(Cell Singaling) in 3% milk/TBS/0.1% Tween 20, 1∶2000 goat anti-rabbit IgG (Santa Cruz Biotechnology) as secondary antibody in 3% milk/TBS/0.1% Tween 20 was used to incubate membranes at room temperature for 2 h. Proteins were detected by DAB detection system (Zhongshan Golden Bridge Bio. Co., Beijing China). The densitometry of the bands were analyzed using Gelpro3.5 software.

### Statistics

Data are expressed as mean ± SEM, GraphPad (SanDiego, CA) were used for statistical analysis. Significant difference among multiple groups with one variant was determined by one-way *ANOVA*, every two groups were then compared using *Newman-Keuls*. The Student *t* test was used for comparisons between two groups. A two-tailed *P* value <0.05 was considered statistically significant.

## Results

### Expression of CD68 in Paired Lung Cancer, Adjacent Tumor Tissues and Surrounding Non-tumorous Lung Tissue from Patients

CD68 is the specific surface marker of macrophages. As expected, CD68 were mainly detected in cytoplasm of macrophages with brown staining (**Figure 1**). The number of cell with positive CD68 staining in adjacent lung cancer tissue was 97.49±2.54, which was higher than that in lung cancer tissues (11.78±1.37) and non-tumorous lung tissues (52.14±1.85), and the differences were significant (n = 67, *P*<0.05).

### Clinical Implication of CD68 Expression in Adjacent Lung Cancer Tissue

The correlation was studied between CD68 high expression in adjacent lung cancer tissue and clinicopathologic characteristics of lung cancer including age, gender, differentiation of tumor cell, metastasis to lymph node, TNM stage and tumor invasion to pleura (**Table 1**). It was shown that the number of CD68 positive staining macrophages was closely associated with TNM stage (*P* = 0.001), metastasis to lymph node (*P* = 0.001) and tumor invasion to pleura (*P* = 0.001).

### Tumorigenic Transformation of BEAS-2B Cells Treated by CTPE

The tumorigenicity of BEAS-2B cells treated with DMSO, CTPE, B(a)P, or Co-culture/CTPE was evaluated through examination of cell cycle, karyotype and colony formation in soft agar at passages 10, 20 and 30.

Tumorigenicity is characterized of growth promotion, which can be determined using analysis of cell cycle by flow cytometry. As shown in **Figure 2**, the percentage of the S phase cells at passage 10 increased in CTPE group and Co-culture/CTPE group; At passage 20, the percentage of cells in S phase in Co-culture/CTPE group was significantly higher than that of BEAS-2B cells alone exposed to CTPE or B(a)P. At passage 30, the percentage of the S phase cells in the Co-culture/CTPE group remained the highest among all groups.

Aneuploidy including hypodiploid (<2n) and hyperdiploid (>2n∼<4n) is a common early feature of cancers and has been associated with tumorigenesis and tumor progression. As shown in **Figure 3**, the number of cells with aneuploidy in total 100 BEAS-2B cells in Co-culture/CTPE group at passage 10 was greater than that of DMSO group, but there was no difference compared to CTPE group. At passage 20 and 30, the rates of cells with aneuploidy in 100 BEAS-2B cells in Co-culture/CTPE group were the highest among these four groups.

The results from soft agar assay demonstrated that the number of colony and clongenicity percentage of BEAS-2B cells (**Figure 4** **–** **5**) in co-culture group were not increased at passage 10, but were significantly increased at passage 20, or passage 30 compared to those of other three groups (*P*<0.05).

### Tumor Xenograft Growth in Nude Mice

Tumor formation in nude mice resembles tumorigenicity *in vivo* (n = 6/group). BEAS-2B cells without treatment were used as the negative control (blank); BEAS-2B cells treated by DMSO were used as vehicle control. 30 days following cell transplantation, there was no tumor formation in blank, DMSO, or CTPE group at passage 20; However, tumors were observed on the back neck of nude mice transplanted with CTPE-treated BEAS-2B cells of passage 30 (the first tumor was observed on the 15^th^ day after transplantation), and the mixture of CTPE-treated BEAS-2B cells at passage 30 and THP-1 cells (two tumors were observed on the 10^th^ day after transplantation) (**Figure 6**). Tumor growth curve demonstrated that the average volume of tumors from CTPE-induced passage 30 BEAS-2B/THP-1 cells increased compared to the same passage of BEAS-2B cells alone exposed to CTPE at different observation time points (*P*<0.05) (**Figure 7**).

### mRNA and Protein Levels of NF-κB in BEAS-2B Cells of Passages 10, 20 and 30

**Figure 8** showed that in the cells of passage 10, NF-κB mRNA levels were significantly increased in CTPE, Co-culture/CTPE or B(a)P groups compared to that of DMSO group. At passages 20 and 30, NF-κB mRNA levels in Co-culture/CTPE group was the highest among all the groups. Similar results were observed with NF-κB protein expression at passages 10, 20 and 30 BEAS-2B cells (**Figure 9**).

## Discussion

Tumor-associated macrophages (TAMs), which have an M2 macrophage phenotype, are the most abundant immune cells in the tumor microenvironment and play mostly pro-tumoral role. Increased TAMs are frequently [26], [27], although not always [28], correlated with a bad prognosis. And there are some reports demonstrating that infiltrating TAMs could promote cancer invasion and metastasis [29]. TAMs infiltrated in breast tumor stroma, not in tumor nest, are positively related to breast tumor metastasis [26], [30]; Similarly, TAMs infiltrated in the invasive front are associated with improvement in hepatic metastasis in colon cancer [31]; and TAMs could provide a favorable microenvironment for non-small lung cancer invasion and progression [18]. In this study, we observed that the expression levels of CD68+ TAMs were increased in adjacent lung tumor tissue and correlated with lung cancer progression and metastasis, which was consistent with these observations. In addition, the following studies have also indicated the relationship between TAMs and carcinogenesis. For example, TAMs infiltration was found in a mouse model of pancreatic tumor carcinogenesis induced by the expression of oncogenic *Kras^G12D^* [32]. Infiltrating TAMs could produce a proliferation inducing ligand (APRIL) on direct stimulation with Helicobacter pylori (Hp) to promote the initiation of gastric lymphoma [33]. To our knowledge, this study is the first of its kind to show that TAMs can promote lung carcinogenesis.

The occurrence and development of cancer involves cell cycle disorganization, leading to an uncontrolled cellular proliferation. [34]. Aneuploidy, a state of abnormal chromosomal number, is a hallmark of human carcinogenesis [35]. Emerging evidence has suggested that aneuploidy is a driver of tumor initiation and growth [36], [37]. Loss of growth inhibition is one of most important characteristic of malignant cells which can be represented as spherical colonies in soft agar *in vitro* and neoplastic growth in nude mice *in vivo* [38]. In this study, the tumorigenic function of BEAS-2B cells with or without THP-1 co-culture following CTPE removal was demonstrated by *in vitro* and *in vivo* assays. And we identified that TAMs could effectively promote BEAS-2B cell growth, increase number of cells with aneuploidy, increase colony formation of BEAS-2B cells *in vitro* at 20^th^ and 30^th^ passages. A recent study demonstrated that human alveolar macrophages could promote DNA bulky stable adduct formation in human lung epithelial cells exposed with polycyclic aromatic hydrocarbon (PAHs) component of airborne particulate matter (PM(2.5)), the explanation could be that alveolar macrophages could metabolize PAHs of PM2.5 to highly reactive metabolites through induction of Cytochrome P450 (CYP) 1A1 expression and CYP1A1 catalytic activity [39]. Therefore, TAMs not only initiate tumorigenesis by inducing gene mutation and chromosomal rearrangement or amplification, but also form tumor microenvironment and exert their pro-tumoral role by affecting fundamental aspects of tumor biology. For example, they can enhance angiogenesis and lymphangiogenesis [40], adjust innate and adaptive immunity, and catalyze structural changes of the extracellular matrix (ECM) compartment [41], [42].

In addition to the malignant transformation effect in vitro, THP-1 cells were also shown to enhance tumor formation in nude mice *in vivo*. The fact that THP-1 cells could not form clone themselves [43], [44] suggested that these cells might promote permanent alterations of the physiologic and social behavior of BEAS-2B cells leading to their insensitivity to growth inhibition by high cell density or low serum concentration. And there was a necrotic area on the surface of tumor derived from the mixed BEAS-2B (passage 30) and THP-1 cells treated with CTPE. It has been proposed that accumulation to necrotic regions of tumor was another prominent hallmark of TAMs [42], which may be regulated by hypoxia-inducible factor-1 (HIF-1).

When inflammation meets cancer, NF-κB works as the matchmaker [45]. NF-κB [46] affects most of the processes of initiation of neoplasia and its malignant progression, through self-sufficiency in growth signals, insensitivity to growth-inhibitory signals, evasion of apoptosis, limitation of replicative potential, tissue invasion and metastasis, and sustained angiogenesis [47]. In a mouse model, Greten et al. specifically knocked out IKK gene in intestinal epithelial cell to inactivate NF-κB, and found the incidence of colitis-associated colorectal cancer was reduced by 80% [48], which suggested that activation of NF-κB could promote occurrence of colitis-associated colorectal cancer. In addition, there was evidence implicating NF-κB activation mediated pathogen-induced carcinogenesis. Hepatitis B virus X protein induced hepatocellular carcinoma by activating NF-κB [49]; Human papillomavirus (HPV) has been proposed to promote oral carcinogenesis through activating NF-κB [50]. Furthermore, KRAS mutation was reported to mediate initiation and promotion of endometrial cancer through inducing NF-κB activation [51]. However, there existed inconsistence between the association of NF-κB activation and urethane-induced lung carcinogenesis. For example, one study demonstrated that epithelial NF-κB activation could promotes the initiation of lung cancer by upregulation of anti-apoptosis gene Bcl-2 expression [52]; In contrary, another study revealed that short-term NF-κB inhibitor, bortezomib might promote lung caicinogenesis, but prolonged NF-κB inhibition could enhance chemical-induced initiation of lung cancer by increased expression of inflammatory cytokine/chemokines, such as interleukin-1β, CXCL1 and CXCL2 [53].

We showed here that the increase in gene transcription of NF-κB was in parallel to its protein levels in BEAS-2B cells co-cultured with THP-1 cells at passages 20 and 30, which were associated with genomic instability and increase in cell-growth and colony formation. Thus, we speculated that there must be a close relationship between activation of NF-κB in malignant cells and lung cancer initiation. To verify this hypothesis, we tested the involvement of NF-κB in this study and found that CTPE could activate NF-κB, and THP-1 could further promote the activity of NF-κB. The possible explanation could be that TAMs could release pro-inflammatory cytokines and participate in the formation of tumor microenvironment. For example, TNF-α and IL-1β secreted from macrophages are not only essential in initiation of chronic inflammation, but also associated with the initiation of cancer by activating NF-κB signal pathway [54]. Activation of NF-κB in tumor cells can produce ROS that further induces DNA damage and genomic instability, leading to the activation of pro-tumorigenic transcription factors, such as STAT3 and AP-1. These transcription factors can promote overexpression of anti-apoptosis genes, bcl-x(L) [55] and cell-cycle genes, c-Myc, cyclinD1 [48], and induce tumor cell abnormal growth.

In summary, malignant transformation of BEAS-2B cell induced by CTPE can be an excellent *in vitro* model to study lung cancer pathogenesis. Tumor-promoting inflammation represents a critical step in lung carcinogenesis: chronic and smoldering inflammation induced by tumor-associated macrophages may precede tumor initiation, creating a favorable microenvironment in which cells with cancer-causing mutations thrive. NF-κB activation, as one of the pillars of inflammation, may have a promoting role in the occurrence of lung cancer. A better understanding of the molecular mechanism at early stage of lung carcinogenesis would provide useful information in establishment of novel preventive and therapeutic strategies for high risk people of lung cancer and lung cancer patients.
